# HPV genotyping in head and neck cancer: Insights from a Colombian cohort

**DOI:** 10.1016/j.bjorl.2026.101845

**Published:** 2026-06-16

**Authors:** Sergio D. Cruz-Romero, Andrea Estefanía Ramos, José A. Hakim, Alberto Escallón Cubillos, Ana M. Baldión, Sandra Perdomo, Paula Alejandra Baldión, Paula A. Rodríguez-Urrego

**Affiliations:** a University Hospital Fundación Santa Fe de Bogotá, Pathology Department, Bogotá, Colombia; b Universidad Nacional de Colombia, Faculty of Science, Bogotá, Colombia; c University Hospital Fundación Santa Fe de Bogotá, Head and Neck Surgery, Bogotá, Colombia; d International Agency for Research on Cancer (IARC/WHO), Genomic Epidemiology Branch, Lyon, France; e Universidad Nacional de Colombia, Dental School, Oral Health Department, Bogotá, Colombia

**Keywords:** Human papillomavirus virus, Genotype, Papillomavirus infections, Head and neck squamous cell carcinoma, Colombia

## Abstract

**Objective:**

To describe the frequency and distribution of Human Papillomavirus (HPV) infection in patients with Head and Neck Squamous Cell Carcinoma (HNSCC) treated at a tertiary hospital in Colombia.

**Methods:**

A descriptive cross-sectional study was conducted in 81 patients diagnosed with HNSCC between 2015 and 2021 at Fundación Santa Fe de Bogota, Colombia, as part of the Colombian cohort of the InterCHANGE and HEADSpace study. Formalin-Fixed Paraffin-Embedded (FFPE) tumor samples were analyzed for HPV DNA detection and genotyping. Fifty-nine samples were processed using the HPV-Direct Flow CHIP assay (Master Diagnóstica, Seville, Spain), and 22 with the INNO-LiPA HPV Genotyping Extra assay (Fujirebio, Gothenburg, Sweden). Both techniques identify high- and low-risk HPV genotypes through PCR amplification and reverse hybridization. Immunohistochemistry for p16 was performed in all cases using the anti-p16INK4a (E6H4 clone) antibody, considering strong nuclear and cytoplasmic staining in ≥ 70% of tumor cells as positive. Descriptive statistics included absolute and relative frequencies.

**Results:**

The mean age was 66-years (±11), and 64.2% were male. The oropharynx was the most frequent tumor location (50.6%, n = 41), followed by the oral cavity (35.8%, n = 29) and larynx (13.6%, n = 11). Overall, 51.9% (n = 42) of tumors were HPV DNA-positive. HPV infection was more frequent in oropharyngeal carcinomas (92.7%, n = 38) than in the oral cavity (6.9%, n = 2) or larynx (18.2%, n = 2). HPV-16 was the predominant genotype (79.4%), followed by HPV-35, HPV-18, and HPV-33. p16 overexpression was observed in 49.4% (n = 40) of cases, mainly in oropharyngeal carcinomas (87.8%, n = 36). Among HPV DNA-positive tumors, 92.5% (n = 37) were also p16-positive.

**Conclusions:**

This study demonstrates a high prevalence of HPV, predominantly HPV-16, in oropharyngeal carcinomas compared with other head and neck sites in our cohort. These results provide baseline data for Colombia and highlight the need for multicenter studies including more heterogeneous populations to guide future prevention and control strategies.

**Level of evidence:**

4 (According to the Oxford Centre for Evidence-Based Medicine, 2011).

## Highlights

• Colombian study characterizing HPV genotypes in head and neck cancer.
• Predominance of HPV-associated oropharyngeal squamous carcinomas.
• HPV-16 identified as main genotype with other high-risk variants.
• Strong concordance between HPV DNA and p16 expression confirmed.
• Supports genotype-based HPV surveillance and prevention in Colombia.

## Introduction

Head and Neck Squamous Cell Carcinoma (HNSCC) encompasses malignant neoplasms originating from the squamous epithelium of the head and neck, including the oral cavity, oropharynx, larynx, and nasopharynx.^1^ Collectively, HNSCC ranks among the ten most common cancers, representing approximately 4.5% of all cancer diagnoses worldwide.^2^ An increase in the incidence of HNSCC has been observed globally, mainly associated with tobacco use in Southeast Asia and Human Papillomavirus (HPV) infection in Europe and the United States.^3^ In Colombia, 1494 cases of lip, oral cavity, and pharyngeal cancers were reported between 2007 and 2011.^4^

The primary risk factors for HNSCC have traditionally been prolonged tobacco and alcohol use.^5^ However, other factors such as poor oral hygiene, dietary habits, and viral infections have also been implicated.^6^ Among these, HPV infection has emerged as a major etiologic factor, particularly in Oropharyngeal Squamous Cell Carcinoma (OPSCC).^7^ HPV-associated OPSCC exhibits distinct clinical, demographic, and molecular features and generally confers a more favorable prognosis than HPV-independent tumors.^8^^,^^9^

HPV is a double-stranded DNA virus characterized by its E6 and E7 oncoproteins, which inactivate key tumor suppressors such as p53 and Retinoblastoma protein (pRb), promoting cellular transformation.^10^ More than 200 genotypes have been identified, grouped as High-Risk (HR-HPV) or low-risk (LR-HPV) based on their oncogenic potential.^11^ HR-HPV types, including HPV-16, HPV-18, and others, are most frequently associated with malignant transformation.^12^

In oropharyngeal tumors, p16 Immunohistochemistry (IHC) is widely used as a surrogate marker of transcriptionally active HPV infection, reflecting viral integration and the downstream inactivation of pRb by the E7 oncoprotein.^13^^,^^14^ While p16 overexpression can occur through non-viral mechanisms, strong nuclear and cytoplasmic staining in more than 70% of tumor cells is considered indicative of HPV-driven carcinogenesis.^15^^,^^16^ Outside the oropharynx, p16 expression may lack clinical relevance, but in OPSCC it remains the most reliable indicator for transcriptionally active HPV.^14^^,^^16^

Globally, the prevalence of oral HPV infection varies by region, with rates of 6.6% in the United States and 4.08% in China.^17^, ^18^, ^19^ Among head and neck subsites, OPSCC is the only location where HPV has been clearly recognized as an etiologic factor, with reported concordance rates between p16 expression and HPV DNA or RNA detection ranging from 85% to 95%.^15^^,^^20^ In contrast, the association between HPV and squamous cell carcinomas of the larynx and oral cavity remains controversial, with reported HPV positivity ranging from 0% to 85% in laryngeal tumors and 3% to 10% in oral cavity cancers, suggesting a weaker or incidental role in carcinogenesis.^21^, ^22^, ^23^

In Colombia, a study conducted in four pathology laboratories in Medellín using PCR genotyping reported an overall HPV infection frequency of 18.9% in HNSCC, with HPV detected in 23.9% of oral cavity tumors, 17.5% of laryngeal tumors, and 13.3% of oropharyngeal carcinomas. However, p16 IHC was not performed, limiting conclusions about viral transcriptional activity.^24^

Given the growing relevance of HPV in oropharyngeal carcinoma and the limited data available for the Colombian population, the present study aims to describe the frequency and genotypic distribution of HPV infection in patients with HNSCC across different anatomical sites and to evaluate p16 expression as a surrogate marker of viral integration.

## Methods

This analytical observational cross-sectional study was conducted on patients from the Colombian cohort of the study, recruited at a tertiary hospital in Fundación Santa Fe de Bogotá, Colombia, between 2015 and 2021 corresponding to the Colombian cohort of interCHANGE and HEADspace. A total of 81 patients with confirmed HNSCC diagnosis in different head and neck anatomical subsites and Formalin-Fixed Paraffin-Embedded (FFPE) tissue samples were included. Additionally, information on demographic characteristics, lifestyle, and clinical features was obtained through a structured questionnaire designed for the study. This questionnaire included variables such as sex, age at diagnosis, educational level, tobacco use, alcohol consumption, and sexual behavior (Table 1).

**Table 1:** Comparative demographic and behavioral characteristics of patients with head and neck squamous cell carcinoma by anatomical site (oropharynx vs. oral cavity and larynx).

Category	Subcategory	Oropharynx (*n* = 41)		Oral Cavity (*n* = 29), Larynx (*n* = 11)		Total (*n* = 81)	
		Percentage (%)	Count (n)	Percentage (%)	Count (n)	Percentage (%)	Count (n)
Age	Mean	63 years (±10)		68 years (±11)		66 years (±11)	
Gender	Male	78.04	32	50.00	20	64.20	52
	Female	21.95	9	50.00	20	35.80	29
Education Level	University	48.78	20	65.00	26	56.79	46
	Secondary	14.63	6	15.00	6	14.81	12
	Primary	24.39	10	10.00	4	17.28	14
	Incomplete Primary	7.31	3	10.00	4	8.64	7
	Not available	4.87	2	0	0	4.76	2
Smoking Status	Smokers/Ex-smokers	63.41	26	55.00	23	60.49	49
	Never smoked	36.59	15	45.00	17	39.51	32
Alcohol Consumption	Current drinker/Former drinker	97.56	40	62.50	25	79.01	65
	Never	2.44	1	37.50	15	20.99	16
Number of Sexual Partners	Two or more partners	60.98	25	10.00	4	35.80	29
	Less than two partners	28.83	11	40.00	16	33.33	27
	Not available	12.20	5	50.00	20	30.86	25
Oral sex	Yes	8	19.51	22.50	9	20.99	17
	No	68.29	28	30.00	12	49.38	40
	Not available	12.19	5	47.50	19	29.63	24

### HPV genotyping

A total of 81 FFPE HNSCC samples were analyzed. DNA extraction for all samples was performed using the Quick-DNA & RNA FFPE MiniPrep kit (Zymo Research, Irvine, CA, USA). Of these, 59 samples were genotyped at the Pathology Department of Fundación Santa Fe de Bogotá using the HPV-Direct Flow CHIP assay (Master Diagnóstica, Seville, Spain), which identifies 36 HPV genotypes (18 high-risk and 18 low-risk) through multiplex PCR, reverse hybridization, and automated reading. The remaining 22 samples were processed following the HEADLACE protocol, which involves proteinase K-SDS digestion and organic extraction, and were analyzed at the Innovation in Cancer Laboratory at ICESP (São Paulo, Brazil) using the INNO-LiPA HPV Genotyping Extra assay based on the principle of reverse hybridization after highly sensitive PCR amplification (Fujirebio, Gothenburg, Sweden), capable of identifying 32 HPV genotypes.

### Immunohistochemistry (IHC)

IHC for p16 was performed in all the samples to evaluate the sobreexpression of the p16INK4a protein. The slides were deparaffinized with heat for 1 h, the antigen retrieval and the immunostaining process were performed in the BenchMark ULTRA system (Roche, Basel, Switzerland), the primary antibody anti-p16INK4a, clone E6H4 (Roche, Basel, Switzerland), was used as the detection system UltraView Universal DAB (Roche, Basel, Switzerland), and subsequently counterstaining was performed with hematoxylin. According to the 5th edition of the WHO Classification of Head and Neck Tumours, a positive result for p16 was defined as strong nuclear and cytoplasmic staining (3+) in more than 70% of tumor cells. p16-positive Oropharyngeal Squamous Cell Carcinoma (OPSCC) was classified as HPV-associated OPSCC, serving as a surrogate marker of HPV DNA integration.^25^

### Statistical analysis

The data obtained were organized into a database using Excel (Microsoft Office 2010) and analyzed with the PAST 4.3.1 statistical package. A descriptive statistical analysis was performed, presenting absolute and relative frequencies for categorical variables, and measures of central tendency and dispersion (mean and standard deviation) for continuous variables. The results are expressed as percentages and means to summarize the demographic and clinical characteristics of the study population. This study was reviewed and approved by the institutional ethics committees of both participating institutions. Ethical approval was granted by the Ethics Committee of the of the Facultad de Odontología, Universidad Nacional de Colombia. Recruitment procedures and informed consent protocols were approved by the Ethics Committee of the Fundación Santa Fe de Bogotá. All procedures involving human participants were conducted in accordance with the ethical principles of biomedical research outlined in the Declaration of Helsinki and complied with Colombian regulations for health research, specifically Resolution nº 8430 of 1993.

## Results

### Demographics

A total of 81 patients diagnosed with Head and Neck Squamous Cell Carcinoma (HNSCC) were included. The mean age was 66-years (±11, range 55–74), and 64.2% were men. The Oropharynx (OPSCC) was the most frequent site (50.6%), followed by the Oral Cavity (OC = 35.8%) and Larynx (LC = 13.6%). Within OPSCC, the most common subsites were the palatine tonsil (53.7%) and the base of the tongue (29.3%), followed by metastatic SCC of unknown primary (9.8%), posterior pharyngeal wall (4.9%), and soft palate (2.4%).

Patients with OPSCC were slightly younger (mean 63 ± 10 years) than those with OC or LC (mean 68 ± 11 years). In OPSCC, 48.8% were under 65-years and 51.2% were 65 or older. Men predominated (78.0%), while in OC–LC, sex distribution was equal (50% each). Regarding education, 48.8% of OPSCC patients had university studies, 14.6% secondary, 24.4% primary, and 7.3% incomplete primary education. In the OC–LC group, 65.0% had university education, 15.0% secondary, and 20.0% primary or incomplete primary schooling.

### Tobacco and alcohol use

Tobacco use was common across all tumor sites, though more frequent in OPSCC (63.4%) than in OC–LC (55.0%). Among OPSCC patients, 24.4% smoked fewer than five cigarettes per day, 39.0% smoked more than five, and 36.6% were non-smokers. Similar patterns were observed for smoking duration, with 43.9% smoking for more than 10-years, 19.5% for fewer than 10-years, and 36.6% never smoking. In OC–LC, 40.0% reported smoking for over 10-years, 17.5% for fewer than 10-years, and 42.5% never smoked. When smoking status after diagnosis was considered for the overall cohort, 39.5% of patients had never smoked, 59.3% were former smokers, and 1.2% continued smoking after the diagnosis.

Alcohol consumption was nearly universal among OPSCC patients (97.6%), compared with 62.5% in OC–LC. Most OPSCC patients reported drinking on fewer than two days per week (63.4%) or on two or more days weekly (35.0%). In the OC–LC group, 37.5% reported drinking fewer than two days per week and 25.0% two or more days, while 37.5% denied alcohol use. Considering alcohol consumption after diagnosis in the entire cohort, 48.1% of patients continued drinking alcohol, 32.1% were former drinkers, and 19.8% reported never consuming alcohol.

### Sexual behavior

Information on sexual practices was available for 57 patients (70.4%). In OPSCC, 60.9% reported having two or more sexual partners, 28.8% fewer than two, and 12.2% did not provide this information. Among OC–LC patients, 10.0% reported two or more partners, 40.0% fewer than two, and 50.0% did not respond. Oral sex practices were reported by 19.5% of OPSCC and 22.5% of OC–LC patients, denied by 68.3% and 30.0%, respectively, with missing data in 12.2% and 47.5%.

### Clinical outcomes

Staging was determined according to the 8th edition of the AJCC. Among oral cavity carcinomas, 51.7% were classified as stage I–II and 48.3% as stage III–IV. In OPSCC, 73.2% of cases were diagnosed at early stages (I–II), while 24.4% were advanced (III–IV). All laryngeal carcinomas with available staging data were classified as stage I, except for one case staged as III.

At the three-year follow-up, overall mortality was 29.6% and recurrence occurred in 13.6% of patients. When stratified by primary site, mortality was 25.9% in oral cavity carcinomas and 18.2% in laryngeal carcinomas. Within the oropharynx, mortality differed according to HPV status, occurring in 26.8% of HPV-associated OPSCC and 7.3% of HPV-independent OPSCC; among deaths in HPV-associated OPSCC, 90.9% occurred in patients with a history of smoking. Recurrence was not observed in oral cavity carcinomas and occurred in 3.5% of laryngeal carcinomas. Within OPSCC, recurrence was documented in 19.5% of HPV-associated cases and 2.4% of HPV-independent cases; among recurrent HPV-associated OPSCC, 75.0% had a history of smoking.

Regarding therapeutic management in patients with unfavorable clinical evolution, treatment strategies varied according to the primary tumor site and pattern of recurrence. In OPSCC, systemic treatments included platinum-based chemotherapy combined with taxanes (carboplatin-paclitaxel), epidermal growth factor receptor inhibitor (cetuximab), and immune checkpoint inhibitors such as nivolumab or pembrolizumab, administered either as monotherapy or in combination with chemotherapy and/or radiotherapy, depending on prior treatments and disease extent. In contrast, surgical salvage was indicated only in selected cases with localized and resectable recurrence, particularly in non-oropharyngeal sites.

### HPV DNA infection

Overall, 51.9% of tumors were positive for HPV DNA, of which 81.0% were single infections and 19.0% co-infections (Table 2). HPV-16 was the predominant genotype, present in 79.4% of single infections, followed by HPV-35, HPV-18, HPV-33, and HPV-31. Seven cases showed co-infection with two genotypes and one with three, most commonly HPV-16 and HPV-35. No low-risk genotypes were detected in single infections, though HPV-44 and HPV-55 appeared in co-infections with HPV-16.

**Table 2:** Frequency of HPV infection by location (HPV DNA).

Location	No infection		Single Infection		Co-infection		Total
	(*n*)	(%)	(n)	(%)	(*n*)	(%)	(*n*)
Oral Cavity	27	33.33	2	2.47	0	0.00	29
Larynx	9	11.11	2	2.47	0	0.00	11
HPV-independent OPSCC	3	3.70	1	1.23	1	1.23	5
HPV associated OPSCC	0	0.00	29	35.80	7	8.64	36
Total	39	48.15	34	41.98	8	9.88	81

### p16 Immunohistochemistry

p16 overexpression was detected in 49.4% of tumors, most frequently in OPSCC (87.8%), followed by laryngeal (18.2%) and oral cavity carcinomas (6.9%). Among the 42 HPV DNA-positive cases, 92.5% were also p16-positive, indicating strong concordance between HPV DNA detection and p16 expression. Of these, 36 were HPV-associated OPSCC and one was laryngeal carcinoma. Similarly, 87.8% of HPV DNA–negative tumors were also p16-negative, confirming the high agreement between absence of HPV infection and lack of p16 expression (Table 3).

**Table 3:** Distribution of identified HPV genotypes (HPV DNA).

Genotype (HPV)	Single infection				Co-infection			
	Oral cavity	Larynx	HPV-independent OPSCC	HPV-associated OPSCC	Oral cavity	Larynx	HPV-independent OPSCC	HPV-associated OPSCC
High-risk								
HPV-16	0	1	0	26	0	0	1	7
HPV-35	0	0	0	2	0	0	1	5
HPV-33	0	1	0	1	0	0	0	0
HPV-18	1	0	1	0	0	0	0	1
HPV-31	0	1	0	0	0	0	0	0
Low-risk								
HPV-55	0	0	0	0	0	0	0	1
HPV-44	0	0	0	0	0	0	0	1

## Discussion

The findings of this study provide valuable insights into the prevalence and genotypic characterization of HPV in patients with head and neck squamous cell carcinoma (HNSCC) from Colombia, expanding current knowledge about the distribution of HPV infection and p16 expression in our population. The oropharynx was the most frequent tumor location (50.62%), and most oropharyngeal tumors were HPV-associated, as defined by p16 overexpression (87.80%). A high frequency of HPV DNA detection was observed in oropharyngeal carcinomas (92.68%), with HPV-16 as the predominant genotype. The strong concordance between HPV DNA and p16 expression reinforces the utility of p16 immunohistochemistry as a reliable surrogate marker for transcriptionally active HPV, as recommended by the WHO classification.^25^

HPV-associated OPSCC (p16-positive) was more frequently diagnosed at early stages (I–II), while HPV-independent OPSCC (p16-negative) predominated in advanced stages (III–IV). This pattern aligns with established evidence that HPV-associated OPSCC constitutes a biologically distinct entity with different molecular mechanisms and better clinical outcomes than HPV-independent tumors.^15^^,^^16^ Although these findings are consistent with global trends, they should be interpreted cautiously given the descriptive nature of the study and the sample size (Table 4).

**Table 4:** p16 IHC/HPV DNA correlation by site.

Category	Oral cavity		Oropharynx		Larynx		Total	Total
	(*n*)	(%)	(*n*)	(%)	(*n*)	(%)	(*n*)	(%)
p16+/HPV+	0	0.00	36	97.30	1	2.70	37	100
p16-/HPV+	2	40.00	2	40.00	1	20.00	5	100
p16+/HPV-	2	66.67	0	0.00	1	33.33	3	100
p16-/HPV-	25	69.44	3	8.33	8	22.22	36	100

HPV infection plays a central etiological role in oropharyngeal HNSCC among our cohort of Colombian patients. In this cohort, 87.8% of oropharyngeal carcinomas were HPV-associated, a frequency comparable to that reported in other high-prevalence regions. Studies from Brazil have shown rates up to 80% of HPV-associated OPSCC,^26^ while the National Cancer Database in the United States reports 68%. In Northern Europe, HPV prevalence ranges from 55% in Denmark to 70% in Sweden, and even higher in Lebanon (85%).^27^ These findings indicate that the Colombian epidemiological profile mirrors global patterns, placing the country among regions with high HPV-associated OPSCC prevalence.

Overall, HPV DNA was detected in 92.68% of oropharyngeal carcinomas, contrasting with the lower detection rates observed in oral cavity (6.90%) and laryngeal tumors (18.18%). HPV-16 was the predominant genotype, followed by HPV-35, HPV-33, and HPV-18. Similar to previous Colombian studies,^24^ our data confirm the dominance of HPV-16 in OPSCC, consistent with global series showing 88% of HPV-positive cases linked to this genotype.^28^ The identification of additional high-risk genotypes such as HPV-35 and HPV-33 supports the inclusion of a broader spectrum of HPV types in molecular surveillance and epidemiological monitoring of head and neck cancer.^29^^,^^30^^,^^31^

The demographic profile of our cohort also corresponds with previous descriptions of HPV-associated OPSCC. Most patients were male (78.0%) with a mean age of 63-years, situating them between middle-aged and older adults. This profile parallels recent reports showing that HPV-associated OPSCC increasingly affects men in their sixth and seventh decades of life, often characterized by higher educational levels and behavioral risk factors such as alcohol consumption and sexual practices.^15^^,^^18^^,^^19^ Although infection was more frequent among men, this should be interpreted with caution, given that HNSCC generally exhibits an approximate 2:1 male-to-female ratio.^32^

Age distribution in our study also reflects the global epidemiological shift toward older men. Patients with OPSCC had a nearly even division between those younger and older than 65-years, consistent with international findings reporting an increasing incidence among men over 65 while maintaining elevated rates in younger adults. Studies from the United States and Europe have documented a rise in the median age at diagnosis from the early 50 s to the late 50 s over the past two decades, likely reflecting generational changes in sexual behavior.^31^

Information on sexual behavior was available for 57 patients (70.4%). Among OPSCC cases, 60.9% reported having two or more sexual partners, a pattern more common in HPV-associated tumors (88.0%) compared with HPV-independent ones (12.0%). Although the study did not seek statistical associations, these findings align with prior research showing a 1.41-fold increased risk of HPV infection among individuals with multiple sexual partners.^33^ Moreover, sexual behavior, particularly the number of lifetime oral sex partners, has been strongly correlated with HPV-associated OPSCC incidence, helping to explain the predominance of these tumors among men.^31^

The concordance observed between HPV DNA detection and p16 expression (92.5%) underscores the reliability of combining these two methods to confirm HPV involvement in oropharyngeal carcinogenesis. In our series, 36 cases corresponded to HPV-associated OPSCC and one to a laryngeal carcinoma, reinforcing current diagnostic standards in the WHO 5th edition classification.^25^

From a public health standpoint, the recognition of HPV as an etiologic agent in OPSCC has driven preventive measures, particularly vaccination programs. Initially developed for cervical cancer prevention in women, HPV vaccines were later extended to men to reduce viral transmission and prevent HPV-related malignancies in both sexes.^34^^,^^35^ Despite proven efficacy, male vaccination coverage remains suboptimal ‒ only about one in eleven men has initiated the HPV vaccine series in the United States.^36^

In Colombia, HPV vaccination has been expanded to include boys aged 9–14 years, complementing the existing program for girls aged 9–17 years. The national immunization program currently employs Gardasil®9, a nonavalent vaccine protecting against nine HPV genotypes (6, 11, 16, 18, 31, 33, 45, 52, and 58). Considering the genotype distribution observed in head and neck carcinomas, preventive and therapeutic approaches ‒ including vaccination and molecular surveillance ‒ should be tailored to the epidemiological profile of oropharyngeal cancer rather than extrapolated from cervical cancer models.^32^

Although HPV plays a well-established etiologic role in oropharyngeal squamous cell carcinoma, its contribution to carcinogenesis and disease progression in non-oropharyngeal sites, particularly the oral cavity and larynx, remains less clearly defined. Experimental and molecular studies have demonstrated that HPV infection may participate in early oncogenic events in oral epithelial cells through the activity of viral oncoproteins E6 and E7, which interfere with cell cycle regulation, apoptosis, and genomic stability.^37^ Additionally, HPV DNA has been detected in oral and laryngeal squamous cell carcinomas, suggesting a potential cofactor role in tumor initiation or progression, especially in the presence of traditional carcinogens such as tobacco and alcohol.^37^^,^^38^ However, unlike OPSCC, the etiological fraction attributable to HPV in oral cavity and laryngeal carcinomas appears to be substantially lower, and its clinical and prognostic relevance remains controversial.

A limitation of this study is the potential for selection bias due to the relatively small sample size and the characteristics of the population, as it represents a convenience sample from a single private hospital primarily serving patients with private insurance. These factors may limit the generalizability of the findings to the national level. Future multicenter studies including patients from different regions and healthcare systems in Colombia are warranted to allow broader demographic and clinical comparisons.

## Conclusion

This descriptive study provides an updated overview of HPV infection in head and neck squamous cell carcinoma in Colombia. A high frequency of HPV-associated OPSCC was observed, characterized by p16 overexpression and predominance of the HPV-16 genotype. The demographic and clinical characteristics of this subgroup, predominantly affecting men in their sixth and seventh decades of life and those with higher educational levels, are consistent with previously reported profiles of HPV-associated OPSCC. The prevalence observed in this cohort is similar to that reported in Brazil (80%) and higher than that described in the United States (68%), Denmark (55%), and Sweden (70%), and comparable to the high rates reported in Lebanon (85%), placing Colombia among countries with a high frequency of HPV-associated oropharyngeal carcinoma. These findings provide baseline information for the Colombian population and emphasize the need for multicenter studies that include patients from diverse regions and healthcare systems, as well as longitudinal evaluations in vaccinated populations, to strengthen epidemiological surveillance and prevention strategies for HPV-associated head and neck cancer.

## ORCID IDs

Sergio D. Cruz-Romero: 0000-0001-9472-7515

Andrea Estefanía Ramos: 0009-0005-6511-8362

José A. Hakim: 0009-0004-7750-4888

Alberto Escallón Cubillos: 0009-0004-3853-7767

Ana M. Baldión: 0000-0002-3785-2237

Sandra Perdomo: 0000-0001-7740-8141

Paula A. Rodríguez-Urrego: 0000-0003-3693-7690

## Ethics approval and consent, data

All procedures performed in this study involving human participants were conducted in accordance with the local research guidelines of the Universidad Nacional de Colombia and adhered to the principles outlined in the Declaration of Helsinki. The institutional ethics committee of the Facultad de Odontología at the Universidad Nacional de Colombia approved the study under document B.CIEFO-192-2022. The recruitment and informed consent protocols were approved by the Comité de Ética de la Fundación Santa Fe de Bogotá (CCEI-2424-2014). Informed consent was obtained from each individual included in the study.

## Declaration of Generative AI and AI-assisted technologies in the writing process

During the preparation of this work, the authors used ChatGPT 4.0 for English translation and proofreading. After utilizing this tool, the authors reviewed and edited the content as needed and took full responsibility for the content of the published article.

## Funding

This work was funded by the “Convocatoria nacional para el fortalecimiento de la formación a través del apoyo a proyectos de investigación, creación artística e innovación de la Universidad Nacional de Colombia 2022–2024” [HERMES:57550], and by the International Agency for Research on Cancer (IARC) through the HEADSpace Project funded by the European Union’s Horizon 2020 Framework Programme (grant nº 825771).

## Data availability statement

The data that supports the findings of this study are available from the corresponding author, upon reasonable request.

## CRediT authorship contribution statement

**Sergio D. Cruz-Romero:** Conceptualization, Methodology, Validation, Formal analysis, Investigation, Data curation, Writing - original draft, Visualization, Funding acquisition. **Andrea Estefanía Ramos:** Conceptualization, Methodology, Validation, Formal analysis, Investigation, Data curation. **José A. Hakim:** Resources. **Alberto Escallón Cubillos:** Resources. **Ana M. Baldión:** Writing - review & editing, Supervision, Funding acquisition. **Sandra Perdomo:** Writing - review & editing, Visualization, Funding acquisition. **Paula Alejandra Baldión:** Conceptualization, Methodology, Validation, Formal analysis, Investigation, Data curation, Writing - review & editing, Visualization, Funding acquisition. **Paula A. Rodríguez-Urrego:** Conceptualization, Methodology, Validation, Formal analysis, Investigation, Data curation, Writing - review & editing, Visualization, Funding acquisition.

## Declaration of competing interest

The authors declare no conflicts of interest related to this study. Where authors are identified as personnel of the International Agency for Research on Cancer/World Health Organisation, the authors alone are responsible for the views expressed in this article and they do not necessarily represent the decisions, policy or views of the International Agency for Research on Cancer/World Health Organisation.

## References

[1] Johnson D.E., Burtness B., Leemans C.R., Lui V.W.Y., Bauman J.E., Grandis J.R. (2020). Head and neck squamous cell carcinoma. Nat Rev Dis Primers.

[2] Barsouk A., Aluru J.S., Rawla P., Saginala K., Barsouk A. (2023). Epidemiology, risk factors, and prevention of head and neck squamous cell carcinoma. Med Sci (Basel).

[3] Gormley M., Creaney G., Schache A., Ingarfield K., Conway D.I. (2022). Reviewing the epidemiology of head and neck cancer: definitions, trends and risk factors. Br Dent J.

[4] López A.F., Sanabria Quiroga Á.E. (2020). Características clínicas y administrativas que se relacionan con el retraso en el diagnóstico y tratamiento de los pacientes con cáncer de cabeza y cuello. Rev Colomb Cir.

[5] Sun Z., Sun X., Chen Z., Du J., Wu Y. (2022). Head and neck squamous cell carcinoma: risk factors, molecular alterations, immunology and peptide vaccines. Int J Pept Res Ther.

[6] Aghiorghiesei O., Zanoaga O., Nutu A. (2022). The world of oral cancer and its risk factors viewed from the aspect of microRNA expression patterns. Genes (Basel).

[7] Lechner M., Liu J., Masterson L., Fenton T.R. (2022). HPV-associated oropharyngeal cancer: epidemiology, molecular biology and clinical management. Nat Rev Clin Oncol.

[8] Cantrell S.C., Peck B.W., Li G., Wei Q., Sturgis E.M., Ginsberg L.E. (2013). Differences in imaging characteristics of HPV-positive and HPV-negative oropharyngeal cancers: a blinded matched-pair analysis. AJNR Am J Neuroradiol.

[9] World Health Organization (WHO) (2024).

[10] Katirachi S.K., Grønlund M.P., Jakobsen K.K., Grønhøj C., Von Buchwald C. (2023). The prevalence of HPV in oral cavity squamous cell carcinoma. Viruses.

[11] Pešut E., Đukić A., Lulić L. (2021). Human papillomaviruses-associated cancers: an update of current knowledge. Viruses.

[12] Michaud D.S., Langevin S.M., Eliot M. (2014). High-risk HPV types and head and neck cancer. Int J Cancer.

[13] Venuti A., Paolini F. (2012). HPV detection methods in head and neck cancer. Head Neck Pathol.

[14] Mehanna H., Taberna M., Von Buchwald C. (2023). Prognostic implications of p16 and HPV discordance in oropharyngeal cancer (HNCIG-EPIC-OPC): a multicentre, multinational, individual patient data analysis. Lancet Oncol.

[15] Grønhøj Larsen C., Gyldenløve M., Jensen D.H. (2014). Correlation between human papillomavirus and p16 overexpression in oropharyngeal tumours: a systematic review. Br J Cancer.

[16] Lewis J.S., Thorstad W.L., Chernock R.D. (2010). p16-positive oropharyngeal squamous cell carcinoma: an entity with a favorable prognosis regardless of tumor HPV status. Am J Surg Pathol.

[17] Roman B.R., Aragones A. (2021). Epidemiology and incidence of HPV-related cancers of the head and neck. J Surg Oncol.

[18] Giuliano A.R., Felsher M., Waterboer T. (2023). Oral human papillomavirus prevalence and genotyping among a healthy adult population in the US. JAMA Otolaryngol Head Neck Surg.

[19] Yu S., Zhu Y., He H. (2023). Prevalence and risk factors of oral human papillomavirus infection among 4212 healthy adults in Hebei, China. BMC Infect Dis.

[20] Lim Y.X., D’Silva N.J. (2024). HPV-associated oropharyngeal cancer: in search of surrogate biomarkers for early lesions. Oncogene.

[21] Onerci Celebi O., Sener E., Hosal S., Cengiz M., Gullu I., Guler Tezel G. (2018). Human papillomavirus infection in patients with laryngeal carcinoma. BMC Cancer.

[22] Wang H., Wei J., Wang B. (2020). Role of human papillomavirus in laryngeal squamous cell carcinoma: a meta-analysis of cohort studies. Cancer Med.

[23] de Sanjosé S., Brotons M., Pavón M.A. (2018). The natural history of human papillomavirus infection. Best Pract Res Clin Obstet Gynaecol.

[24] Quintero K., Giraldo G.A., Uribe M.L. (2013). Human papillomavirus types in cases of squamous cell carcinoma of head and neck in Colombia. Braz J Otorhinolaryngol.

[25] World Health Organization Classification of Tumours Editorial Board (2024).

[26] Silveira H.A., Almeida L.Y., Carlos R. (2022). Human papillomavirus co-infection and survival in oral and oropharyngeal squamous cell carcinoma: a study in 235 Brazilian patients. Auris Nasus Larynx.

[27] Carlander A.F., Jakobsen K.K., Bendtsen S.K. (2021). A contemporary systematic review on repartition of HPV-positivity in oropharyngeal cancer worldwide. Viruses.

[28] Tagliabue M., Mena M., Maffini F. (2020). Role of human papillomavirus infection in head and neck cancer in Italy: the HPV-AHEAD study. Cancers (Basel).

[29] Kokkinis E., Bastas N.S., Mega I., Tsironis C., Lianou A.D. (2024). Association of HPV with oral and oropharyngeal cancer: current evidence. Maedica (Bucur).

[30] Gillison M.L., Broutian T., Pickard R.K. (2012). Prevalence of oral HPV infection in the United States, 2009–2010. JAMA.

[31] Ferris R.L., Westra W. (2023). Oropharyngeal carcinoma with a special focus on HPV-related squamous cell carcinoma. Annu Rev Pathol Mech Dis.

[32] Barsouk A., Saginala K., Rawla P. (2022). Global epidemiology of cancers of the lip, oral cavity, and pharynx. Eur J Cancer Prev.

[33] Pauli S., Kops N.L., Bessel M. (2022). Sexual practices and HPV infection in unvaccinated young adults. Sci Rep.

[34] Athanasiou A., Bowden S., Paraskevaidi M. (2020). HPV vaccination and cancer prevention. Best Pract Res Clin Obstet Gynaecol.

[35] Diana G., Corica C. (2021). Human papilloma virus vaccine and prevention of head and neck cancer: what is the current evidence?. Oral Oncol.

[36] El Hussein M.T., Dhaliwal S. (2023). HPV vaccination for prevention of head and neck cancer among men. Nurse Pract.

[37] Santacroce L., Di Cosola M., Bottalico L. (2021). Focus on HPV infection and the molecular mechanisms of Oral carcinogenesis. Viruses.

[38] Yang D., Shi Y., Tang Y. (2019). Effect of HPV infection on the occurrence and development of laryngeal cancer: a review. J Cancer.

